# Supplementary material for: 3D Printing of High Viscosity Reinforced Silicone Elastomers
Source: Polymers (Basel). 2021 Jul 8;13(14):2239. doi: 10.3390/polym13142239 (PMC8309234; doi:10.3390/polym13142239)
Supplement: Supplementary file 1 [file polymers-13-02239-s001.zip › SI Polymers_Silicones Oakdale/Polymers Supporting Information.pdf]

polymers

MDPI

Supporting Information

3D Printing of High Viscosity Reinforced Silicone Elastomers

Nicholas Rodriguez [1,2,†], Samantha Ruelas [3,†], Jean-Baptiste Forien [3], Nikola Dudukovic [1], Josh DeOtte [1], Jennifer Rodriguez [3], Bryan Moran [1], James P. Lewicki [1], Eric B. Duoss [1] and James S. Oakdale [3,*]

[1] Materials Engineering Division, Lawrence Livermore National Laboratory, 7000 East Avenue, Livermore, CA 94550, USA; nick.rodriguez@utexas.edu (N.R.); dudukovic1@llnl.gov (N.D.); deotte1@llnl.gov (J.D.); moran5@llnl.gov (B.M.); lewicki1@llnl.gov (J.P.L.); duoss1@llnl.gov (E.B.D.)
[2] Department of Mechanical Engineering, The University of Texas at Austin, 204 E. Dean Keeton Street, Austin, TX 78712, USA
[3] Materials Science Division, Lawrence Livermore National Laboratory, 7000 East Avenue, Livermore, CA 94550, USA; ruelas7@llnl.gov (S.R.); forien1@llnl.gov (J.-B.F.); rodriguez96@llnl.gov (J.R.)
* Correspondence: oakdale1@llnl.gov; 925-424-4157.
† N.R. and S.R. contributed equally.

Citation: Rodriguez: N.; Ruelas, S.; Forien, J.-B.; Dudukovic, N.; DeOtte, J.; Rodriguez, J.; Moran, B.; Lewicki, J.P.; Duoss, E.B.; Oakdale, J.S. 3D Printing of High Viscosity Reinforced Silicone Elastomers. *Polymers* **2021**, *13*, 2239. https://doi.org/10.3390/polym13142239

Academic Editor: Jia Min Lee

Received: 7 June 2021
Accepted: 2 July 2021
Published: 8 July 2021

Publisher's Note: MDPI stays neutral with regard to jurisdictional claims in published maps and institutional affiliations.

Copyright: © 2021 by the authors. Licensee MDPI, Basel, Switzerland. This article is an open access article distributed under the terms and conditions of the Creative Commons Attribution (CC BY) license (http://creativecommons.org/licenses/by/4.0/).

Table of Contents

Figure S1: Measured Intensity at the build plate as function of aperture diameter. (pg SI-2)
Table S1: Solubility of photo-initiator components in TEMQ-1. (pg SI-2)
Figure S2: Viscosity of VTS-2 + MFS-1 as a function of silica content. (pg SI-2)
Figure S3: ASTM D638 tensile testing for TEFS-1 through TEFS-4 compared to 'bulk' specimen. (pg SI-3)
Figure S4.: Tensile data for TEFS-1 specimens. (pg SI-3)
Figure S5: Tensile data for TEFS-2 specimens. (pg SI-3)
Figure S6: Tensile data for TEFS-3 specimens. (pg SI-4)
Figure S7: Tensile data for TEFS-4 specimens. (pg SI-4)
Figure S8: DSC analysis of TEFS1-4. (pg SI-5)
Figure S9: Resin Shear modulus as a function of filler concentration (wt%). (pg SI-5)
Figure S10: Tensile data for TEFS-5 specimens. (pg SI-6)
Figure S11: Tensile data for TEFS-6 specimens. (pg SI-6)
Figure S12: Tensile data for TEMQ-1 specimens. (pg SI-6)
Figure S13: Tensile data for TEMQ-2 specimens. (pg SI-7)
Figure S14: Tensile data for TEMQ-3 specimens. (pg SI-7)
Figure S15: Tensile data for TEMQ-4 specimens. (pg SI-7)
Figure S16: Layer thickness as a function of photo-absorber. (pg SI-8)
Figure S17 Stress vs strain data for a TEFS-3 octet truss lattice compressed to 87% strain. (pg SI-8)

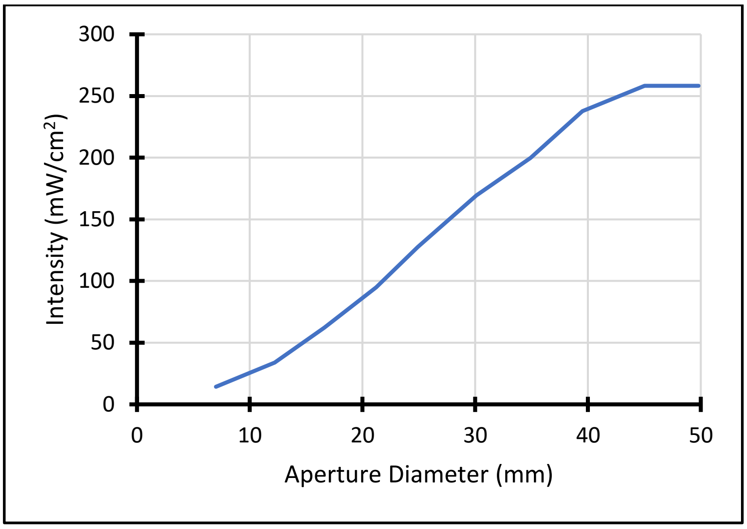

Figure S1. Measured intensity at the build plate as a function of aperture diameter. An aperture diameter of 12.2 mm was used for the curing discussed in this research with a resulting maximum intensity of 34 mW/cm^2 at the build plane.

Table S1. Solubility of photo-initiator components in TEMQ-1. Compounds were dissolved in 0.25 mL THF and added to 2.5 grams of TEMQ-1 and mixed via planetary centrifugal Thinky mixing. The resulting mixture was left open to the atmosphere to allow the THF to escape and solubility was checked after seven days. A lack of solubility was noted for samples that either turned cloudy or formed a noticeable precipitate.

Reagent	Role	Solubility in PDMS (wt%)
Isopropylthioxanthone (ITX)	Sensitizer	0.15
2-ethylhexyl 4-(dimethylamino)benzoate (EHDA)	Initiator	2.4
2-(2H-benzotriazol-2-yl)-4,6-di-tert-pentylphenol (BTA)	Absorber	0.24
2,5-Bis(5-tert-butyl-benzoxazol-2-yl)thiophene	Absorber	0.003
4-methoxyhydroquinone (MEHQ)	Inhibitor	0.20
Ethyl (2,4,6-trimehylbenzoyl) phenylphosphinate	Initiator	0.12
9,10-diethoxyanthracene	Sensitizer	0.06
Camphorquinone	Sensitizer	1.2

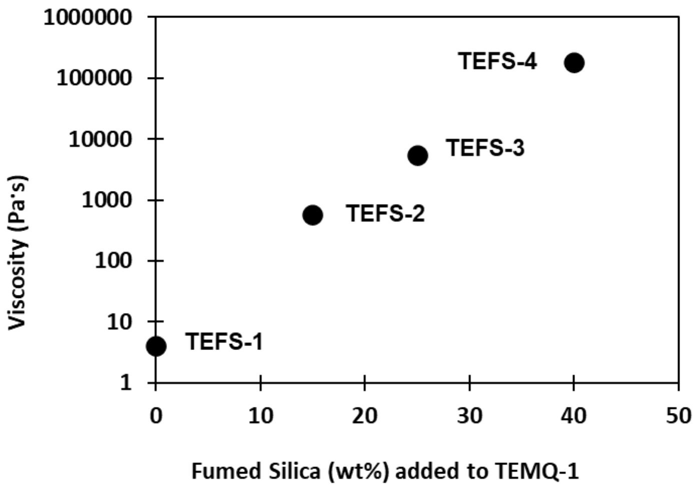

Figure S2. Viscosity of VTS-2 + MFS-1 as a function of silica content. TEMQ-1 = 0 phr. TEMQ-2 = 10 phr. TEMQ-3 = 25 phr. TEMQ-4 = 33 phr.

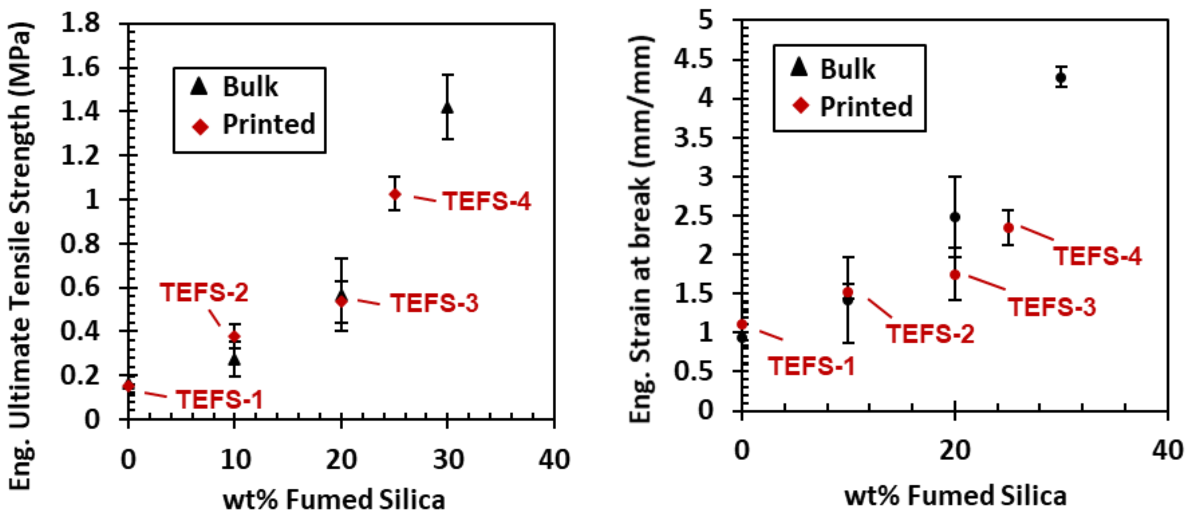

Figure S3. ASTM D638 tensile testing for TEFS-1 through TEFS-4 compared to 'bulk' specimen. TEFS samples were 3D printed (red diamond) in the XY orientation at 405 nm 34 mW/cm². Bulk samples from which ASTM D638 specimen were die-cut, were cast in 3 mm thick sheet and cured under a 405 nm 250 mW/cm² LED flood lamp.

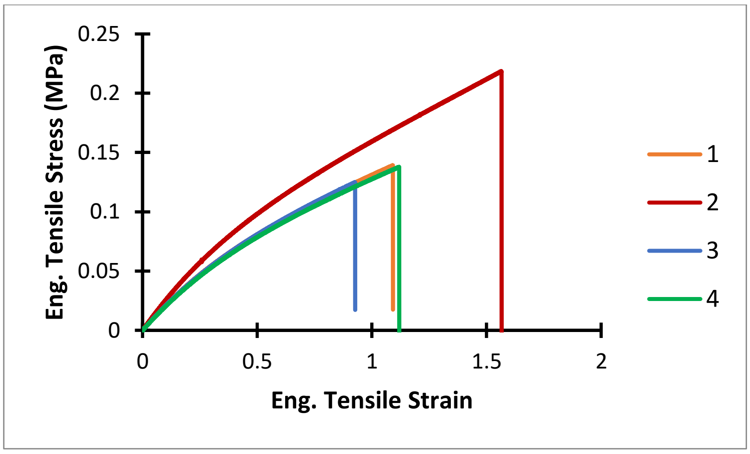

Figure S4. Tensile data for TEFS-1 specimens.

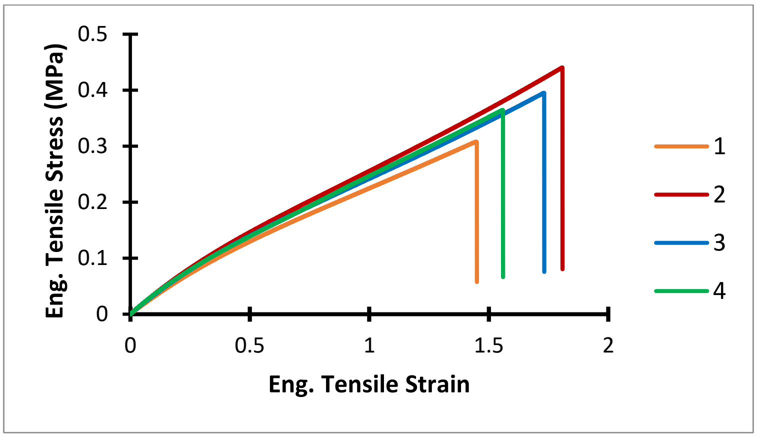

Figure S5. Tensile data for TEFS-2 specimens.

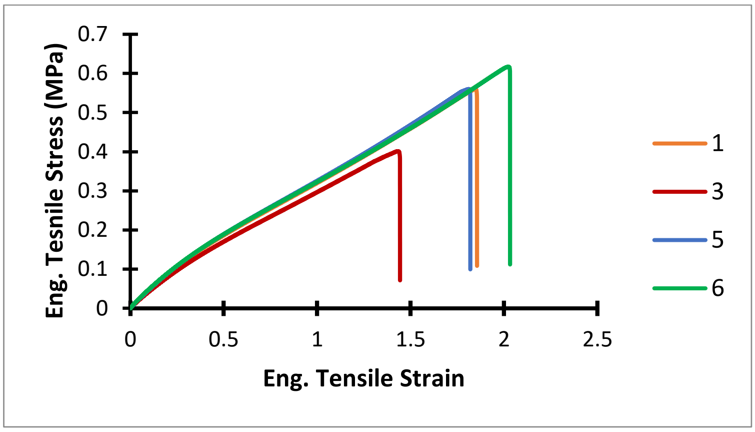

Figure S6. Tensile data for TEFS-3 specimens.

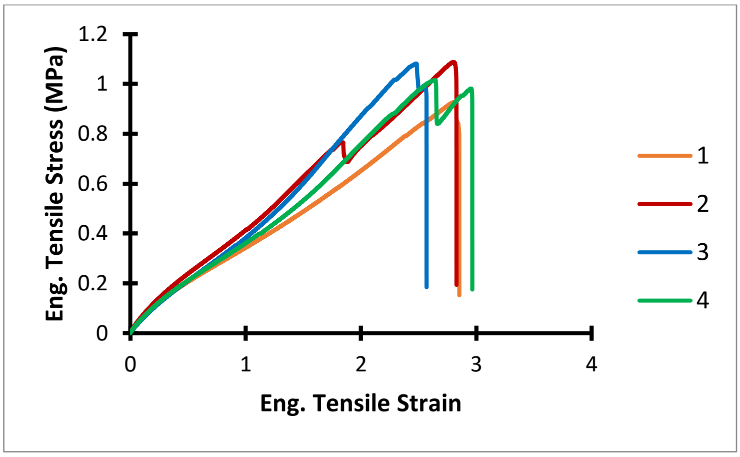

Figure S7. Tensile data for TEFS-4 specimens.

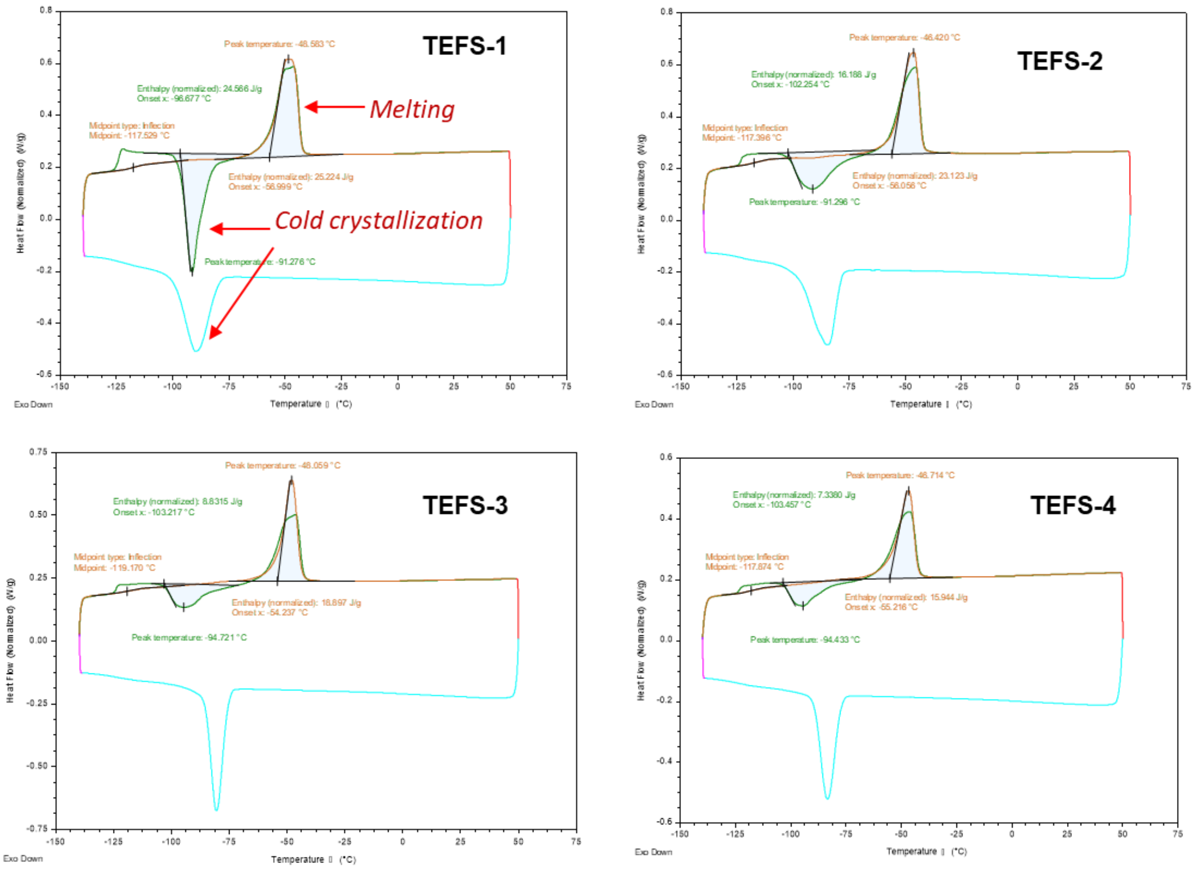

Figure S8. DSC analysis of TEFS1-4. Green trace corresponds to heating from -147 °C to 50 °C at 10 °C per min following quench freezing for one minute. The Light blue trace corresponds to cooling from 50 °C to -147 °C at 10 °C per min. The orange trace corresponds to a final heat through the entire range of temperatures observed.

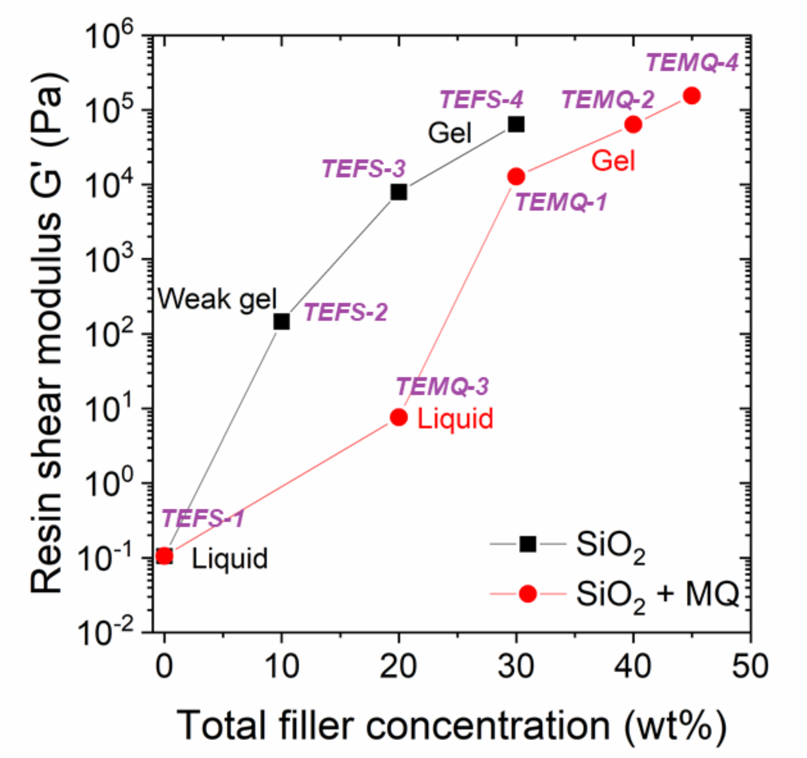

Figure S9. Resin shear modulus as a function of filler concentration (wt%).

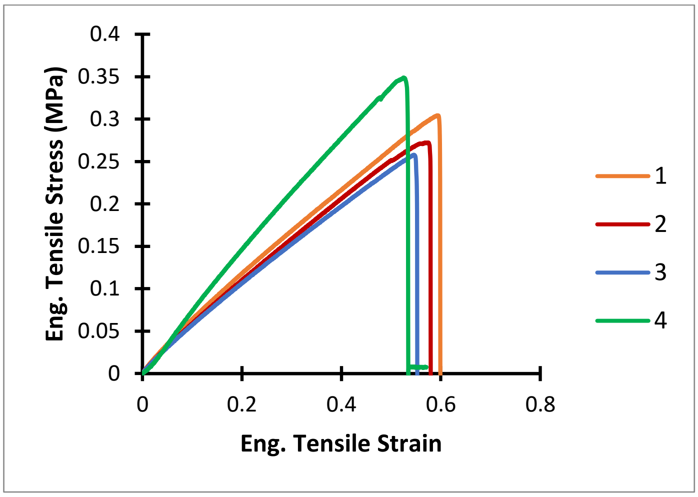

Figure S10. Tensile data for TEFS-5 specimens.

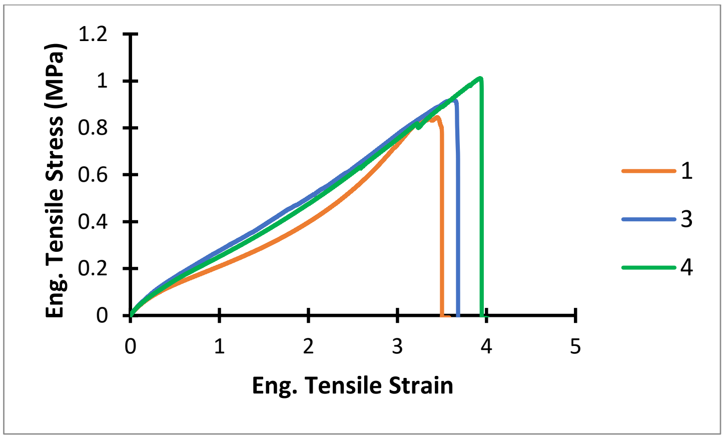

Figure S11. Tensile data for TEFS-6 specimens. Specimen 2 slipped during the course of the experiment—no conclusive data was recorded.

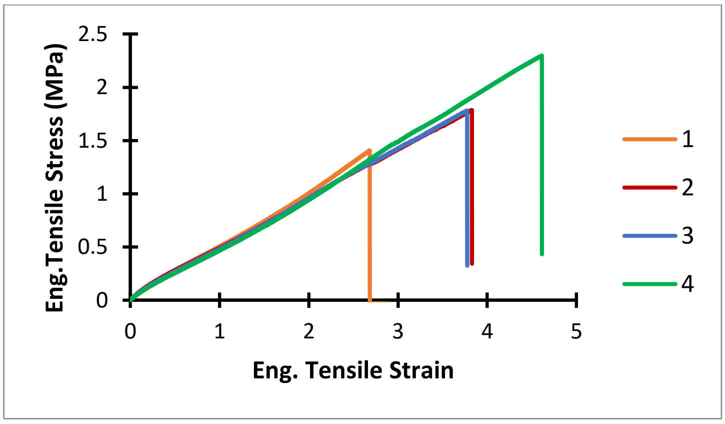

Figure S12. Tensile data for TEMQ-1 specimens.

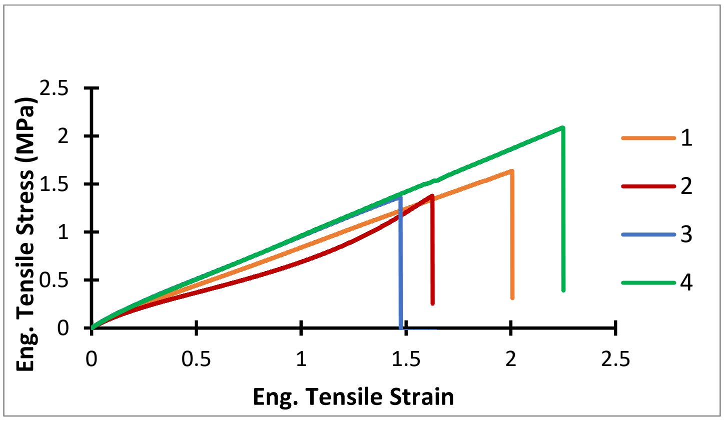

Figure S13. Tensile data for TEMQ-2 specimens.

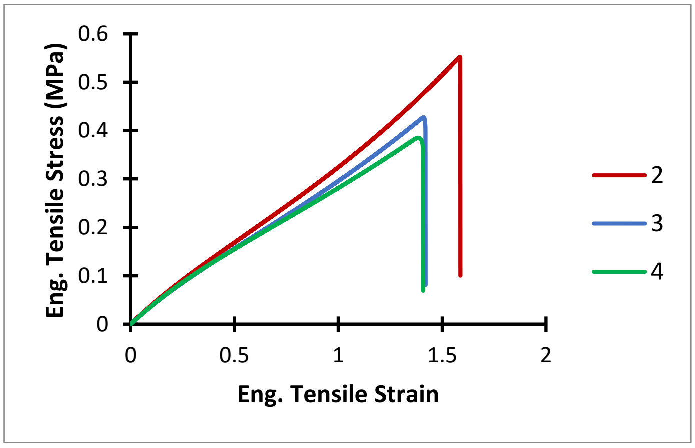

Figure S14. Tensile data for TEMQ-3 specimens. Specimen 1 slipped during the course of the experiment—no conclusive data was recorded.

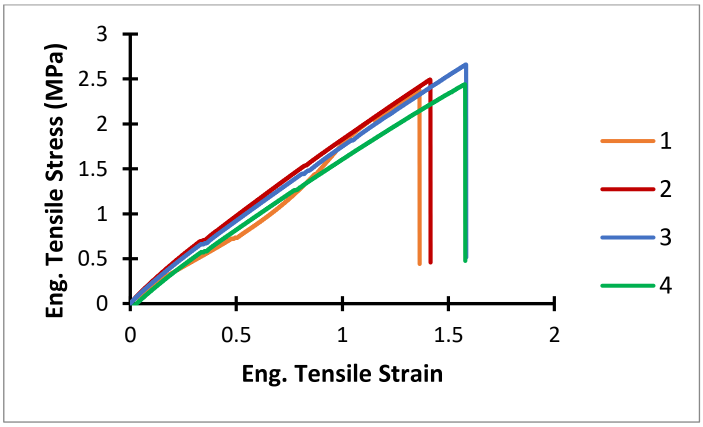

Figure S15. Tensile data for TEMQ-4 specimens.

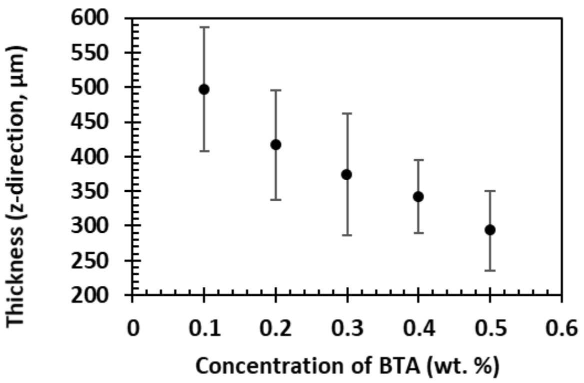

Figure S16. Layer thickness as a function of photo-absorber. BTA concentration was varied in order to decrease layer thickness. Higher concentrations decreased layer thickness by approximately 200 μm.

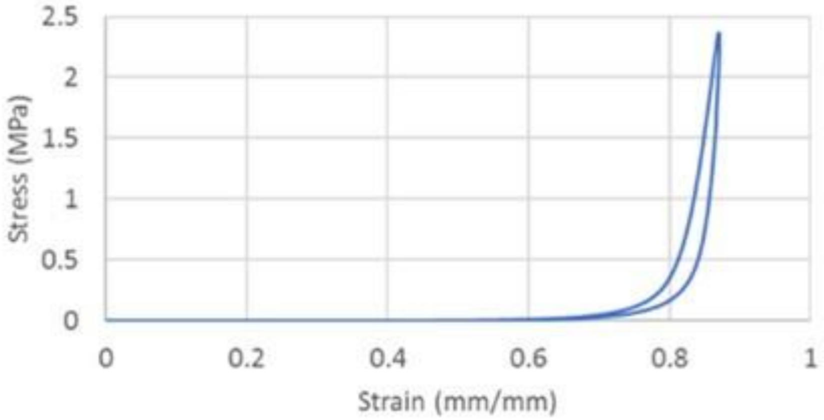

Figure S17. Stress vs strain data for a TEFS-3 octet truss lattice compressed to 87% strain.
